# Physico-chemical properties of *Flacourtia jangomas* seed oil encapsulated with pea protein isolate and maltodextrin

**DOI:** 10.1016/j.fochx.2026.104406

**Published:** 2026-09-04

**Authors:** Thi Thuy Le, Phuong Vy Tra, Ngoc Nhu Huynh Nguyen, Hoang Nhi Tran, Thi Thu Tra Tran, Van Viet Man Le

**Affiliations:** a Ho Chi Minh City University of Technology (HCMUT), 268 Ly Thuong Kiet Street, Dien Hong Ward, Ho Chi Minh City, Viet Nam; b Vietnam National University – Ho Chi Minh City (VNU-HCM), Vo Truong Toan Street, Linh Xuan Ward, Ho Chi Minh City, Viet Nam

**Keywords:** Encapsulation, *Flacourtia jangomas* seed oil, Pea protein isolate, Dextrose equivalent, Freeze drying

## Abstract

This study evaluated the effects of maltodextrin with different dextrose equivalent (DE) and pea protein isolate (PPI) ratios in the wall material on the physicochemical characteristics and storage stability of freeze-dried *Flacourtia jangomas* seed oil (FJSO) powder. Among the formulations tested, the capsules prepared with maltodextrin DE5 and a PPI ratio of 50% exhibited the highest encapsulation yield and efficiency, at 91.86 ± 0.93% and 88.11 ± 0.99%, respectively. Physical properties of oil powders were not significantly affected by the DE of maltodextrin, while particle size decreased with increasing DE. The PPI ratio influenced powder flowability, which was greatest at intermediate PPI levels (33–50%). Compared with the unencapsulated oil, all microcapsule powders showed significantly improved oxidative stability under accelerated storage conditions, with maltodextrin DE5 and a PPI ratio of 50% samples exhibiting the highest stability. Overall, the suitable PPI-maltodextrin wall system showed potential for producing stable seed oil capsules with enhanced oxidative protection.

## 1 Introduction

Edible vegetable oils play an essential role in human nutrition by providing a concentrated source of metabolic energy (Abdel-Naem et al., 2024), supplying essential fatty acids (Kamińska et al., 2025), and facilitating the absorption of fat-soluble vitamins (Nguyen et al., 2019). Conventionally, these oils are classified into seed oils (e.g., soybean, sunflower, canola oils) and fruit oils (e.g., coconut, olive, avocado oils) (Cicero et al., 2018). Among these, canola and sunflower oils dominate in terms of both production volume and nutritional value (Zio et al., 2025). Nevertheless, in response to the escalating global demand for sustainable food systems, the exploration of novel vegetable oil sources has become an urgent research priority. Recent studies have highlighted the growing commercial interest in unconventional oil sources, including oils derived from fruit, vegetable, and herb seeds (Konopka et al., 2023). This has led to increased attention on fruit seed oils, obtained from by-products such as grape, pomegranate, passion fruit, and blackberry seeds (Konopka et al., 2023). These oils are rich in bioactive compounds, such as tocopherols, phytosterols, and polyunsaturated fatty acids (PUFAs). It is reported that red raspberry seed oil contains up to 85% PUFAs (54% linoleic acid and 32% α-linolenic acid), while blackcurrant seed oil comprises approximately 81.5% PUFAs (48% linoleic acid and 30% α- and γ-linolenic acid) (Jurgoński et al., 2018).

*Flacourtia jangomas* (Lour.) Raeusch, commonly known as Hong Quan, is a tropical fruit-bearing plant belonging to the Salicaceae family, native to South Asia. The fruits are ellipsoid, subglobose berries, 1.5 to 2.5 cm in diameter, dark red or purple to almost black, like cherries, and containing multiple small, stony seeds, with a taste ranging from sour to sweet (Pambou-Tobi et al., 2025). Previous studies have examined the proximate composition and antioxidant activity of *Flacourtia jangomas* fruit (Tan et al., 2023) and the pharmacological properties of its seeds (Ripa et al., 2024). However, the nutritional composition and functional properties of oil extracted from *Flacourtia jangomas* seeds have not yet been reported. In contrast, previous studies on seed oils from other Salicaceae species have demonstrated their potential as valuable sources of functional lipids. For example, seed oil from *Idesia polycarpa*, a species in the Salicaceae family, has been characterized for its quality and fatty acid profile, with a high linoleic acid content of 78.76% (Wen et al., 2022). Considering the nutritional potential of fruit seed oils from the Salicaceae family, FJSO may also represent a promising source of functional lipids, although its fatty acid composition and bioactive properties remain to be elucidated. Despite their favorable fatty acid profile and health-promoting properties, fruit seed oils are highly prone to oxidative deterioration due to their elevated PUFA content. Lipid oxidation during storage can result in hydroperoxide formation, nutritional losses, undesirable flavor development, and overall quality degradation (Liaotrakoon et al., 2013), highlighting the need for effective encapsulation technologies to improve oil stability and preserve bioactive compounds throughout processing and storage.

Encapsulation has been developed as a method to protect fatty acids and volatile compounds in edible oils by enclosing oil droplets (core material) within a surrounding wall or supporting matrix (wall material), thereby forming small capsules (Fang & Bhandari, 2010). Encapsulation technology, which began with emulsifying lipids in a solution of wall materials, followed by freeze-drying, is widely used for vegetable oils, such as buriti oil (Pereira de Oliveira et al., 2022), walnut oil (Wang et al., 2024), and chia seed oil (Anand et al., 2024). Freeze-drying is a specialized batch process that preserves heat-sensitive PUFAs by sublimating frozen emulsions under reduced pressure. Despite technical challenges, freeze-drying offers superior nutrient retention and oxidative stability compared to spray-drying, achieving encapsulation efficiencies of up to 99% in various studies (Pattnaik & Mishra, 2022).

Various amphiphilic food-grade biopolymers, such as proteins, carbohydrates, or mixtures of them, have been used as primary wall materials for lipid encapsulation (Gentile, 2020). Proteins stabilize oil droplets through their high surface activity and subsequently form the protective wall matrix during emulsion formation (McClements et al., 2007). However, natural proteins are unstable at the oil/water interface and cannot provide sufficient stability for the emulsion (Yang et al., 2024a). While carbohydrates have high water solubility and enhance emulsion stability by forming a structured network within the continuous phase, they help to maintain the integrity of the core material when combined with proteins. Therefore, protein-carbohydrate complexes have been developed as preferred wall materials to overcome these limitations, leading to significant improvements in oil encapsulation efficiency (Piornos et al., 2017).

Pea protein isolates, a type of vegetable protein, have garnered increasing attention from food processors and consumers due to their high nutritional value, affordability, and non-allergenic properties (Bajaj et al., 2015). Despite these advantages, PPIs exhibit pH sensitivity and tend to form high-viscosity solutions at elevated concentrations (Nesterenko et al., 2013). Therefore, these proteins are often combined with maltodextrin to enhance emulsion stability, as proteins facilitate interfacial adsorption and droplet stabilization, whereas maltodextrin enhances matrix formation and contributes to the overall stability of the encapsulation system (Galves et al., 2021). Sundar et al. (2025) reported that the use of maltodextrin improves the emulsifying properties of pea protein compared with pea protein isolates alone in the microencapsulation of niger seed oil (NSO). This method achieved the highest encapsulation efficiency and improved the oxidative stability of NSO (Sundar et al., 2025).

Maltodextrin, derived from starch hydrolysis, is characterized by a DE value below 20. This characteristic confers good solubility, strong heat stability, easy digestibility, low viscosity at high concentrations, and enhanced oxidative stability of core materials (Hu et al., 2024). However, the functionality of maltodextrin is strongly dependent on its DE, which governs key physicochemical properties such as molecular weight, viscosity, hygroscopicity, and glass transition temperature (Mahdavi et al., 2016). Consequently, variations in DE values can markedly influence the physicochemical characteristics and encapsulation performance of microcapsules, including encapsulation efficiency (EE), encapsulation yield (EY), and particle properties (Negrão-Murakami et al., 2017). Previous studies indicate that maltodextrin with varying DE values produces microcapsules with distinct physicochemical characteristics (Turasan et al., 2015) and varying abilities to protect thermolabile compounds during processing and storage (Yorta et al., 2024). Zhu et al. (2022) investigated the effect of maltodextrin with DE values ranging from 10 to 20 on the encapsulation of soybean oil bodies and reported significant differences in encapsulation performance among the tested formulations, with DE10 exhibiting the highest encapsulation efficiency and the lowest surface oil content (Zhu et al., 2022). Despite these findings, most previous studies have focused on spray-drying systems, while the influence of maltodextrin with different DE values on the properties and encapsulation performance of freeze-dried oil microcapsules remains poorly understood. Therefore, investigating the effects of different DE values is essential for understanding carrier functionality and improving the quality and stability of freeze-dried microencapsulated oils.

This study examines the effects of maltodextrin dextrose equivalent values (5, 10, and 15) in combination with pea protein isolate, as well as variations in the pea protein isolate ratios in the carrier matrix, on the encapsulation yield and efficiency, physicochemical properties, and storage stability of freeze-dried *Flacourtia jangomas* seed oil capsules.

## 2 Materials and methods

### 2.1 Materials

*Flacourtia jangomas* fruits were sourced from a fruit farm in Tinh Bien, An Giang Province, Vietnam (10.5996° N, 104.9498° E), and transported to the laboratory on the day of harvest. Upon arrival, fruits were sorted to remove defective specimens and washed with tap water. The fruits were blanched at 90 °C for 1.5 min to reduce microbial contamination, inactivate oxidative enzymes, and soften fruit tissues by partially disrupting cell wall structures, thereby facilitating subsequent processing. After blanching, the fruits were cooled to ambient temperature, milled, and pressed using a filter bag to obtain the pomace. The pomace was dried at 60 °C for approximately 4 h to reduce the moisture content below 12%. Seeds were manually collected from the dried pomace, then ground and passed through a 70-mesh sieve (212-μm pore size) to produce a powder. The powder was packed in aluminum foil bags and stored at −18 °C for use.

The seed powder was added to n-hexane at a 1:8 (*w*/*v*) ratio; lipid extraction was performed at room temperature for 36 h. The liquid fraction was then collected and subjected to solvent removal at 40 °C and 25 mbar using a vacuum evaporator (N-1300E, EYELA, Japan). The obtained FJSO was stored at −18 °C for 24 h prior to encapsulation.

PPI was sourced from Roquette (Lestrem, France). Maltodextrin with different DE values (5, 10, and 15) was obtained from Ambuja (Mumbai, India). All chemical reagents were of analytical grade and were supplied by Sigma-Aldrich (St. Louis, Missouri, United States).

### 2.2 Characterization of *Flacourtia jangomas* seed oil

The fatty acid profile was determined as fatty acid methyl esters (FAMEs) using gas chromatography with a flame ionization detector (GC-FID) (AOAC 920.12 and AOAC 996.06, 2019).

Antioxidant extraction was performed according to the procedure of Tuberoso et al. (2007) with slight modifications. Briefly, FJSO was mixed with an 80% (*v*/v) aqueous methanol at a 1:1 (*v*/v) ratio and shaken at 30 °C for 30 min in a shaker incubator. The mixture was then centrifuged at 700 ×*g* and 4 °C for 15 min using a refrigerated centrifuge (2-16KL Sigma, Osterode am Harz, Germany). The supernatant was collected and diluted to 25 mL with the extraction solvent. Total phenolic content (TPC) was quantified using the Folin–Ciocalteu colorimetric method (Singleton & Rossi, 1965) and expressed as mg gallic acid equivalent (GAE) per 100 g oil.

Antioxidant activities were determined using 2,2-diphenyl-1-picrylhydrazyl (DPPH) and ferric reducing antioxidant power (FRAP) assays; the results were expressed as μmol Trolox equivalents (TE) per 100 g oil (Szydłowska-Czerniak et al., 2008).

Specific density of seed oil was determined using a calibrated pycnometer at 25 °C (AOAC 920.12 and AOAC 996.06, 2019).

Color of FJSO was determined using a CM-3700 A colorimeter (Konica Minolta, Tokyo, Japan) and expressed by L*, a*, and b* parameters in the CIELab color space.

### 2.3 Encapsulation of *Flacourtia jangomas* seed oil by freeze drying

#### 2.3.1 Effects of maltodextrin dextrose equivalent on the encapsulation of *Flacourtia jangomas* seed oil

Encapsulation of FJSO was performed according to the procedure reported by Benito-Román et al. (2020). PPI and maltodextrin with DE5, DE10, and DE15 were used. First, wall solutions containing 10% (*w*/w) total solids were prepared with PPI/maltodextrin mixtures at a 1/1 (*w*/w) ratio by dissolving them in distilled water. Subsequently, the FJSO was added at a 1/2 (*w*/w) core-to-wall ratio, and the emulsion was then homogenized at 300 bars for 10 passes using a high-pressure homogenizer (APV-2000, Poland) to yield an oil-in-water emulsion. The obtained emulsion was transferred to plastic containers and frozen at −50 °C for 3 h, followed by drying at 50 °C for 45 h under 5 Pa vacuum pressure in a freeze-dryer (TOPTION TPV-50F, China) to obtain the FJSO-encapsulated powder. The resulting powders were ground using a mortar, passed through a 70-mesh sieve (212-μm pore size), and stored at −18 °C for subsequent analyses. The samples were labeled PP (PPI was used as wall material), PM5 (PPI combined with maltodextrin of DE5), PM10 (PPI combined with maltodextrin of DE10), and PM15 (PPI combined with maltodextrin of DE15).

#### 2.3.2 Effects of pea protein isolate and maltodextrin ratio on the encapsulation of the *Flacourtia jangomas* seed oil

Based on the results from the previous section, maltodextrin with DE5 was selected for further investigation. The PPI ratio was varied at 25, 33, 50, 67, and 75% (*w*/w) of the total wall material, corresponding to PPI-to-maltodextrin ratios of 1:3, 1:2, 1:1, 2:1, and 3:1, respectively, and were designated as PM25, PM33, PM50, PM67, and PM75, respectively. All remaining procedures were carried out as described in Section 2.3.1.

### 2.4 Characterization of encapsulated oil powder

#### 2.4.1 Encapsulation efficiency and encapsulation yield

Surface oil content (SOC) of the FJSO powder was determined by the procedure described by Young et al. (1993). Total oil content (TOC) of the FJSO powders was determined according to the Velasco method (Velasco et al., 2006). After disruption of the wall matrix with 25% ammonium hydroxide, lipids were extracted sequentially using diethyl ether, followed by petroleum ether (Prasad et al., 2022). EE and EY of the FJSO powder samples were calculated by Eqs. (1) and (2), respectively:

(1) $$\mathrm{EE}\;\left(\%\right)=\frac{\mathrm{TOC}-\mathrm{SOC}}{\mathrm{TOC}}\;x\;100\%$$

(2) $$\mathrm{EY}\left(\%\right)=\frac{\mathrm{TOC}}{\mathrm{IOC}}\;x\;100\%$$

where TOC is the total oil content of FJSO powders (g), SOC is the surface oil content of FJSO powders (g), and IOC is the initial oil content used to prepare the emulsion for encapsulating FJSO (g).

#### 2.4.2 Physico-chemical properties

##### 2.4.2.1 Moisture

Moisture content was quantified by drying a sample (approximately 1–2 g) to constant weight at 105 °C using a fully automatic moisture analyzer (MS 70, A&D Co., Tokyo, Japan).

##### 2.4.2.2 Bulk and tapped density

Bulk density (BD) and tapped density (TD) of the oil powder samples were determined according to the procedure described by Zhu et al. (2022). Density was calculated as the ratio of sample mass to volume and expressed in g/cm^3^.

##### 2.4.2.3 Carr index and Hausner ratio

Carr index (CI) and Hausner ratio (HR) of oil powder samples were calculated according to Eqs. (3), (4), respectively (Zhu et al., 2022).

(3) $$\mathrm{CI}\;\left(\%\right)=\frac{\mathrm{TD}-\mathrm{BD}}{\mathrm{TD}}\times 100$$

(4) $$\mathrm{HR}=\frac{\mathrm{TD}}{\mathrm{BD}}\;$$

##### 2.4.2.4 Particle size distribution

Particle size distribution of the oil powder samples was evaluated using a laser particle size analyzer and Mastersizer Xplorer software (MAZ3000, Malvern Instruments Ltd., UK); the D[4,3] mean diameter and span value were then recorded.

##### 2.4.2.5 Surface morphology by scanning electron microscopy

Surface morphology of the oil powder particles was observed using scanning electron microscopy (SEM). The oil powder samples were mounted on a sample holder using two adhesive tapes and coated with a thin layer of gold under vacuum. The samples were scanned with an electrically accelerated 10 kV beam (Napiórkowska et al., 2023).

##### 2.4.2.6 Fourier transform infrared spectroscopy (FTIR)

FTIR spectra of *Flacourtia jangomas* seed oil, pea protein, maltodextrin, and encapsulated oil powder were determined using a Fourier transform infrared spectrometer (Thermo Scientific Nicolet 6700, USA) at room temperature. All spectra were recorded between 400 and 4000 cm^− 1^ with a resolution of 4 cm^− 1^ (Pereira de Oliveira et al., 2022).

#### 2.4.3 Storage stability test

To accelerate the lipid oxidation reactions, 50 g of both free oil and encapsulated oil powder samples (PP, PM5, PM10, PM15, PM25, PM33, PM50, PM67, and PM75) were sealed in polyethylene (PE) zip-lock bags (100 mm × 150 mm, thickness 45–50 μm) and stored in a climatic chamber (Jeiotech TH3-PE, Korea) at 60 °C and 75% humidity for 16 days (Upadhyay & Mishra, 2016). At days 0, 4, 8, 12, and 16, about 5 g of oil powder samples were taken out from the chamber, and the lipid oxidation characteristics were assessed by determining the Peroxide Value (PV) and Thiobarbituric Acid-Reactive Substances (TBARS). Peroxide value (PV) was measured following a modified version of the methods reported by Yang et al. (2024). Thiobarbituric acid-reactive substances (TBARS) were measured as described by Pu et al. (2011).

### 2.5 Statistical analysis

Each oil powder sample was prepared in three independent batches. The results were presented as mean ± standard deviation. Analysis of variance (ANOVA) was done using Minitab 16 (Minitab LLC, State College, US). Significant differences were determined using Turkey's test at a 5% significance level (*p* < 0.05).

## 3 Results

### 3.1 Characterization of *Flacourtia jangomas* seed oil

The fatty acid profile of FJSO is shown in Table 1.

**Table 1:** Fatty acid composition and properties of *Flacourtia jangomas* seed oil.

Fatty acid composition of *Flacourtia jangomas* seed oil				Properties of *Flacourtia jangomas* seed oil	
Hexanoic acid (C6:0, %)	0.070 ± 0.003	Palmitoleic acid (C16:1, %)	0.252 ± 0.008	Total phenolic content (mg GAE/kg oil)	58.21 ± 3.27
Lauric acid (C12:0, %)	0.029 ± 0.000	Cis-10 heptadecenoic acid (C17:1, %)	0.115 ± 0.002	Antioxidant activity by DPPH (μmol TE/kg oil)	549.62 ± 1.01
Myristic acid (C14:0, %)	0.111 ± 0.003	Cis-9 oleic acid (C18:1, %)	7.533 ± 0.100	Antioxidant activity by FRAP (μmol TE/kg oil)	80.53 ± 1.89
Palmitic acid (C16:0, %)	32.858 ± 0.297	Linoleic acid (C18:2, %)	34.950 ± 0.247	Density (g/cm^3)^	0.92 ± 0.01
Heptadecanoic acid (C17:0, %)	0.300 ± 0.000	Alpha-linolenic acid (C18:3, %)	19.020 ± 0.082	Peroxide value (meq O_2_/kg oil)	7.23 ± 0.46
Stearic acid (C18:0, %)	4.475 ± 0.081	*Unsaturated fatty* *acid (%)*	*61.860 ± 0.269*	TBARS (μmol MDA/kg oil)	0.72 ± 0.00
Arachidic acid (C20:0, %)	0.200 ± 0.000			L*	23.89 ± 0.01
Behenic acid (C22:0, %)	0.106 ± 0.000			a*	1.49 ± 0.01
Saturated fatty acids (%)	*38.147 ± 0.373*			b*	0.71 ± 0.02

Values are means of triplicate ± standard deviation. Values followed by different letter superscripts in the same row differ significantly (*p* < 0.05). Abbreviations: GAE: gallic acid equivalent; DPPH: 2,2-diphenyl-1-picrylhydrazyl; FRAP: ferric reducing antioxidant power; TE: trolox equivalent; TBARS: thiobarbituric acid reactive substances; MDA: malondialdehyde.

The FJSO contained 38.147 ± 0.373% saturated fatty acids, predominantly palmitic acid (32.858 ± 0.297%). In contrast, the unsaturated fatty acid content was approximately 1.6 times higher, with oleic acid (7.533 ± 0.100%), alpha-linolenic acid (19.020 ± 0.082%), and linoleic acid (34.950 ± 0.247%) being the predominant unsaturated fatty acids. Linoleic acid is one of the most important polyunsaturated fatty acids in nutrition because of its role in preventing cardiovascular diseases (Marangoni et al., 2020). Also, linoleic derivatives serve as structural components of the plasma membrane and as precursors of some metabolic regulatory compounds. The ω-6/ω-3 fatty acid ratio is an important indicator of lipid nutritional quality. An ω-6/ω-3 ratio below 4 is generally considered beneficial for cardiovascular health, whereas ratios exceeding 10 have been associated with increased health risks (Simopoulos, 2002). For FJSO, the ω-6/ω-3 ratio of 1.84 indicates a well-balanced fatty acid profile and falls within the recommended limit of 4.0 suggested by the UK Department of Health (Department of Health, 1994). Therefore, consuming FJSO may provide health benefits due to its favorable fatty acid profile.

Phenolic compounds play a crucial role in protecting vegetable oils against oxidation and contribute to various biological activities (Zhong et al., 2025). The total phenolic content of FJSO was 58.21 ± 3.27 mg GAE/kg oil, which is higher than that of blackberry seed oil (31.9 mg GAE/kg oil) (Matei et al., 2023) but much lower than that of raspberry seed oil (89.0 mg GAE/kg oil) (De Filette et al., 2024). The antioxidant capacities of FJSO determined by the DPPH and FRAP assays were 549.61 ± 1.01 and 80.53 ± 1.89 μmol TE/kg oil, respectively, which are higher than those of watermelon seed oil (431 μmol TE/kg oil and 40 μmol TE/kg oil) but lower than those of almond seed oils (662 μmol TE/kg oil and 129 μmol TE/kg oil) (Yao et al., 2019).

The density of FJSO was approximately 0.92 ± 0.01 g/cm^3^, which falls within the typical range for vegetable oils (0.91–0.93 g/cm^3^) (Awogbemi et al., 2019). The peroxide value of the oil was 7.23 ± 0.46 meq O₂/kg, lower than the FAO/WHO limit for freshly extracted vegetable oils (<10 meq O₂/kg) (Singh et al., 2022). Together with the relatively low TBARS value, these results indicate that FJSO was fresh, stable, and showed no signs of oxidative deterioration. The oil exhibited a dark color with a slight red–yellow hue. The specific compounds responsible for the observed coloration were not identified in the present study.

### 3.2 Effects of maltodextrin dextrose equivalent on the *Flacourtia jangomas* seed oil encapsulation

Table 2 illustrates the effects of maltodextrin with varying DE as a wall material on the physico-chemical characteristics of FJSO powder.

**Table 2:** Effects of maltodextrin with varying dextrose equivalents as wall material on the physico-chemical characteristics of *Flacourtia jangomas* seed powder.

		*Flacourtia jangomas* seed oil encapsulation samples with different dextrose equivalents of maltodextrin			
		PP	PM5	PM10	PM15
Encapsulation efficiency (%*w*/w)		84.42 ± 3.6ᵃ	91.86 ± 0.93ᵇ	87.6 ± 3.45ᵃᵇ	88.71 ± 1.23ᵃᵇ
Encapsulation yield (%w/w)		36.9 ± 0.66ᵃ	50.97 ± 1.09ᶜ	43.68 ± 1ᵇ	35.26 ± 0.36ᵃ
Moisture content (%w/w)		5.86 ± 0.01^b^	4.59 ± 0.12ᵃ	4.73 ± 0.3ᵃ	4.72 ± 0.26ᵃ
Mean particle size (μm)		187.3 ± 3.06^d^	112.4.0 ± 4.1ᵃ	96.8.4 ± 5.4ᵇ	65.3 ± 4.2^c^
Span value		1.86 ± 0.04ᵃ	2.05 ± 0.03^b^	2.41 ± 0.06ᶜ	3.08 ± 0.03ᵈ
Bulk density (g/cm^3^)		0.24 ± 0.02ᵃ	0.25 ± 0.02ᵃ	0.24 ± 0.00ᵃ	0.22 ± 0.00ᵃ
Tapped density (g/cm^3^)		0.43 ± 0.07ᵃ	0.46 ± 0.06ᵃ	0.50 ± 0.04ᵃ	0.50 ± 0.04ᵃ
Carr index (%)		44.20 ± 3.30^a^	46.45 ± 2.27^a^	52.96 ± 1.80^b^	56.06 ± 1.71^b^
Hausner ratio		1.80 ± 0.11^a^	1.87 ± 0.08^a^	2.13 ± 0.08^b^	2.28 ± 0.09^b^
Color	L*	80.8 ± 0.9ᵃ	85.2 ± 0.9ᵇ	84.6 ± 0.5ᵇ	85.5 ± 0.5ᵇ
	a*	3.00 ± 0.2ᵇ	1.0 ± 0.3ᵃ	1.2 ± 0.3ᵃ	1.1 ± 0.3ᵃ
	b*	20.0 ± 0.9ᵇ	15.0 ± 1.1ᵃ	16.5 ± 0.6ᵃ	15.7 ± 0.6ᵃ

Values are means of triplicate ± standard deviation. Values followed by different letter superscripts in the same row differ significantly (*p* < 0.05). PP: Pea protein isolate–encapsulated *Flacourtia jangomas* seed oil; PM5: Pea protein isolate–maltodextrin (DE5) encapsulated *Flacourtia jangomas* seed oil; PM10: Pea protein isolate–maltodextrin (DE10) encapsulated *Flacourtia jangomas* seed oil; PM15: Pea protein isolate–maltodextrin (DE15) encapsulated *Flacourtia jangomas* seed oil.

Table 2 indicates that the sample without maltodextrin in the wall material exhibited the lowest EE at 84.42%. The incorporation of maltodextrin with DE values of 10 and 15 did not significantly alter the EE compared to the PP sample. Nevertheless, the sample containing low-DE maltodextrin (PM5) showed a higher EE of 91.86%. Karrar et al. (2021) reported that maltodextrin can effectively enhance interfacial films on the surface of emulsified droplets, thereby reducing oil content on the microcapsule surface after freeze-drying.

The encapsulation yield of the four samples ranged from 35.26% to 50.97%. The lowest values were observed in the PP and MD15 samples, recorded at 36.9% and 35.26%, respectively. As the DE value of maltodextrin was reduced, the encapsulation yield increased. The highest encapsulation yield was obtained for the PM5 sample, reaching 50.97 ± 1.09%. The long-chain structure of maltodextrin fills interstitial voids within the protein matrix, reducing wall porosity. This densification restricts core material diffusion and minimizes surface losses, significantly enhancing encapsulation efficiency (Xiao et al., 2022).

Moisture content plays a pivotal role in oil powder stability by affecting storage shelf life and glass-transition behavior (Zhao et al., 2022). The incorporation of maltodextrin in the wall material slightly reduced the moisture content of the oil powder samples (Table 2). This may be attributed to the ability of maltodextrin to interact with water molecules through hydrogen bonding, reducing the amount of free water remaining in the powder after drying (Mishra et al., 2014).

Particle size is a key factor in assessing the quality of microcapsules, as it directly impacts their stability, functionality, and performance (Zhao et al., 2022). The oil powder samples formulated with PPI and maltodextrin exhibited smaller particle sizes than those formulated with PPI alone. This could be due to the ability of maltodextrin to reduce protein-protein interactions at the molecular level and to decrease the probability of protein aggregation (Liu et al., 2024). However, as the dextrose equivalent of maltodextrin in the wall material increased, the particle size of the capsules tended to decrease. Lower-DE maltodextrin tended to form larger particles due to its longer molecular chains, while higher-DE maltodextrin produced smaller particles owing to shorter chains and lower viscosity (Siemons et al., 2020). The span index, which reflects the uniformity of particle size distribution in encapsulated powders, is lower for a more uniform particle-size distribution. As shown in Table 2, the capsules prepared with PPI as the sole carrier exhibited the lowest span value (1.86 ± 0.04), indicating a more uniform particle-size distribution in the absence of maltodextrin. With increasing DE from 5 to 15, the span index increased by 23.4%, suggesting that lower-DE maltodextrin contributed to improved particle-size uniformity. Previous studies have mainly focused on the role of maltodextrin DE in emulsion stability and spray-drying systems, whereas information regarding its impact on the physicochemical characteristics, particularly particle size, of freeze-dried oil capsules remains limited.

Table 2 also shows that the bulk and tapped densities of the encapsulated samples ranged from 0.220 to 0.245 g/cm^3^ and 0.431–0.502 g/cm^3^, respectively, with no significant differences among samples (*p* > 0.05), suggesting that maltodextrin with different DE values did not significantly affect density of the freeze-dried oil powder. Flowability of the powders was assessed using the CI, and cohesiveness was evaluated using the HR (Korma et al., 2019). The PP sample exhibited the lowest CI (44.20 ± 3.30%) and HR (1.80 ± 0.11) values (*p* < 0.05). Besides, the CI and HR values of the encapsulated oil powder increased with increasing DE of the maltodextrin. This observation is consistent with the findings of Zhu et al. (2022), who reported a similar trend in the microencapsulation of soybean oil using spray drying.

The encapsulated powder with PPI and maltodextrin exhibited higher L^⁎^ value but lower a^⁎^ and b^⁎^ values compared to that with pea protein alone. This may be attributed to the color-dilution effect of the carrier matrix in the presence of maltodextrin since maltodextrin exhibited substantially higher lightness (L^⁎^ = 98.45 ± 0.43) and lower redness and yellowness values (a^⁎^ = −0.08 ± 0.02; b^⁎^ = 0.25 ± 0.06) compared with PPI (L^⁎^ = 83.92 ± 0.36; a^⁎^ = 2.67 ± 0.03; b^⁎^ = 19.52 ± 0.17). Additionally, the presence of maltodextrin lightened the powder by masking the natural pigments of the protein, resulting in reduced color intensity and diminished red and yellow tones (Sundar et al., 2025).

SEM images of the oil powder particles are presented in Fig. 1. The sublimation process during freeze-drying likely contributed to the formation of large and irregularly shaped particles. The PP sample exhibited irregular, rough, and aggregated particles, with an average particle size of 187.3 ± 3.06 μm, suggesting that PPI alone may have been less effective in forming a compact and stable encapsulating matrix under the applied freeze-drying conditions. In contrast, incorporating maltodextrin resulted in smaller capsules (Table 2). Among the maltodextrin-containing formulations, PM15 exhibited the smallest particle size (65.3 ± 4.2 μm), whereas PM5 showed a relatively smoother and more uniform surface with no visible cracks, indicating good film-forming ability and structural stability. The degree of concavity in powder particles has been correlated with the surface oil content, with a higher content of free oil (Sarkar et al., 2016). This may also explain why PM5 had the highest encapsulation efficiency. In contrast, microcapsules prepared with higher-DE maltodextrin (PM10 and M15 samples) exhibited less uniform surfaces, with wrinkles and slight indentations, likely due to non-uniform shrinkage during drying. These results are consistent with those of Zhu et al. (2022), who report that low-DE maltodextrin enhances the integrity and morphological stability of soybean oil microcapsules during spray-drying and storage.

**Fig. 1:**
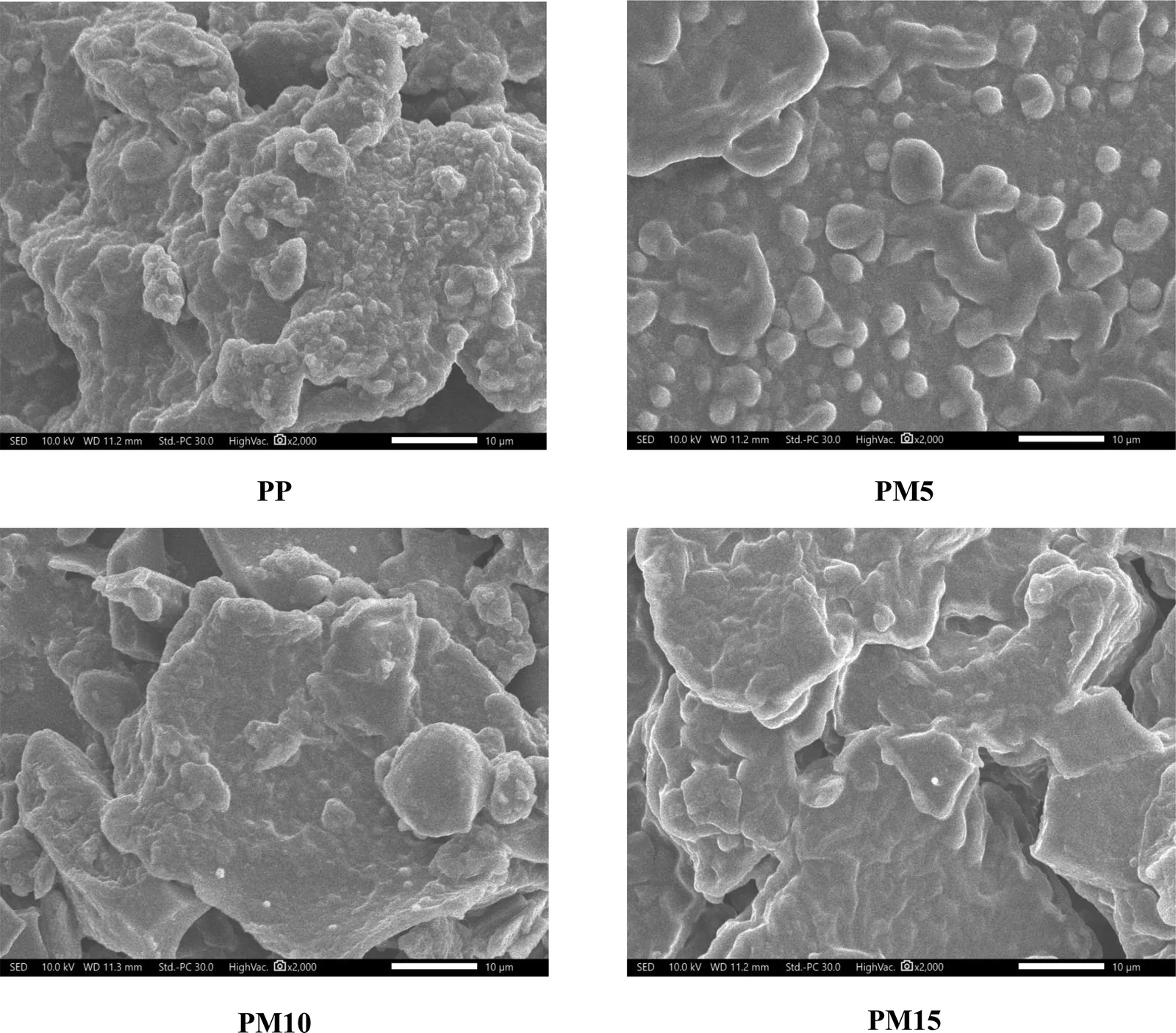
Scanning electron microscopy (SEM) of *Flacourtia jangomas* seed oil encapsulation samples with different dextrose equivalents of maltodextrin as wall material. PP: Pea protein isolate–encapsulated *Flacourtia jangomas* seed oil; PM5: Pea protein isolate–maltodextrin (DE5) encapsulated *Flacourtia jangomas* seed oil; PM10: Pea protein isolate–maltodextrin (DE10) encapsulated *Flacourtia jangomas* seed oil; PM15: Pea protein isolate–maltodextrin (DE15) encapsulated *Flacourtia jangomas* seed oil.

FT-IR spectra of the core, wall materials, and encapsulated powders are presented in Fig. 2. The absorption bands observed in the maltodextrin samples with DE5, 10, and 15 were mainly associated with O—H (3325 cm^−1^), C—H (2919 cm^−1^), C=O (1639 cm^−1^), and C—O (998 cm^−1^) vibrations. Only slight and negligible peak shifts are observed among samples with different DE values, indicating that the polysaccharide backbone remains significantly unchanged. In the PPI spectrum, the amide I band at 1632 cm^−1^ was primarily assigned to C=O stretching, whereas the amide II band at 1528 cm^−1^ is attributed to N—H bending coupled with C—N stretching (Zhu et al., 2022). In the FTIR spectra of FJSO and the encapsulated powders, the absorption bands at around 2993 and 2853 cm^−1^ are attributed to the asymmetric and symmetric stretching vibrations of C—H bonds in aliphatic —CH₃ and —CH₂— groups of fatty acid chains, while the band at 1744 cm^−1^ corresponds to C=O stretching of saturated fatty acids. In addition, the characteristic band observed at 1149 cm^−1^ is attributed to the C—O stretching vibration of ester groups in triacylglycerol, the primary component of FJSO. The presence of these specific lipid peaks, along with the characteristic absorption bands of PP and PM in the microcapsule spectra, confirms the successful entrapment of FJSO within the PPI-maltodextrin wall matrix. Furthermore, in the oil powder spectra, the band around 2300 cm^−1^ corresponds to the asymmetric stretching vibration of the O=C=O bond, which is attributed to atmospheric CO₂ and does not represent structural components of the sample (Schott et al., 2021). The FTIR results suggest that the encapsulation was achieved through physical interactions, with no formation of new chemical bonds between the core and wall materials.

**Fig. 2:**
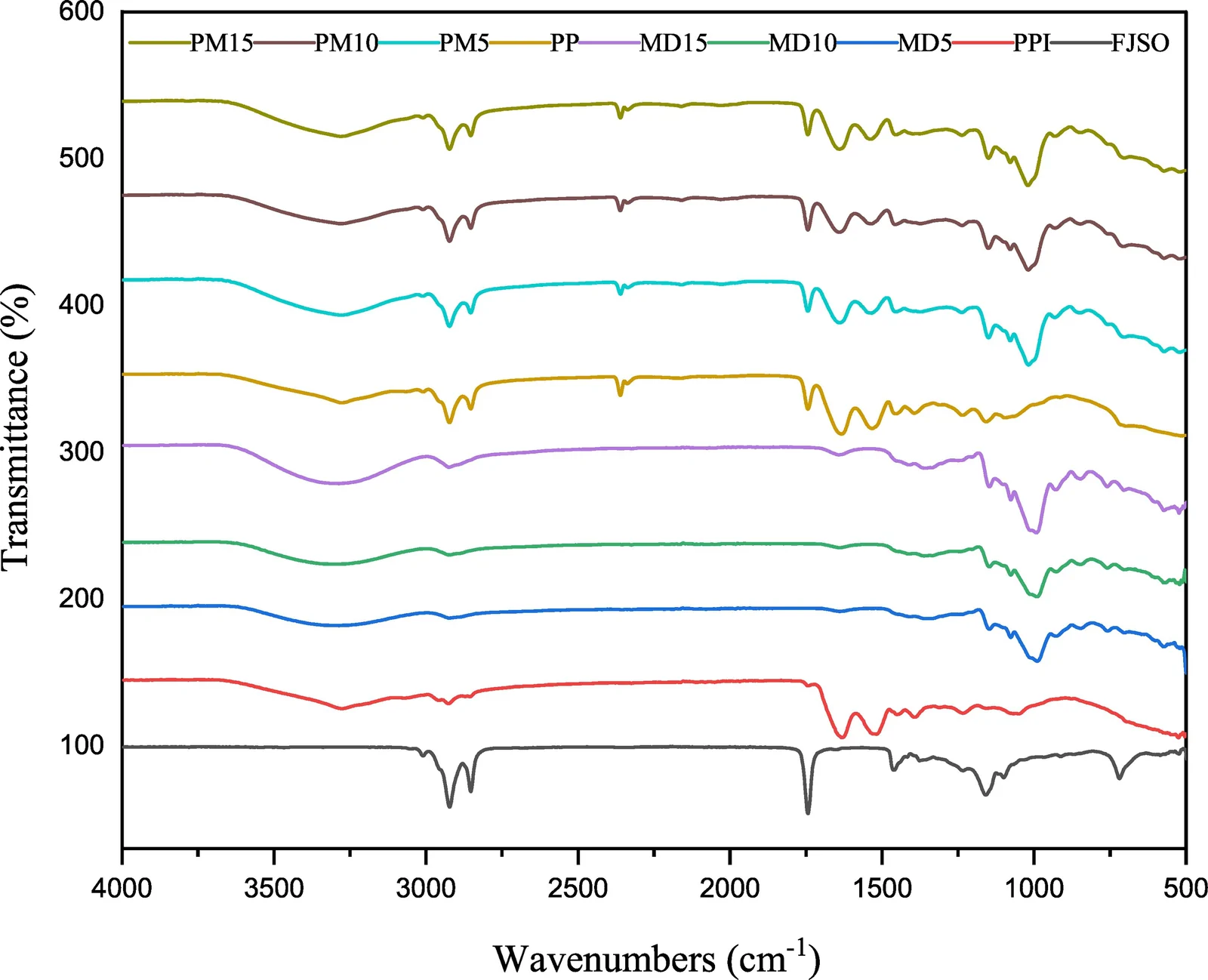
FT-IR spectra of raw materials (*Flacourtia jangomas* seed oil (FJSO), pea protein isolate (PPI), maltodextrin with different dextrose equivalents (MD5, MD10, MD15) and *Flacourtia jangomas* seed oil encapsulation samples. PP: Pea protein isolate–encapsulated *Flacourtia jangomas* seed oil; PM5: Pea protein isolate–maltodextrin (DE5) encapsulated *Flacourtia jangomas* seed oil; PM10: Pea protein isolate–maltodextrin (DE10) encapsulated *Flacourtia jangomas* seed oil; PM15: Pea protein isolate–maltodextrin (DE15) encapsulated *Flacourtia jangomas* seed oil.

During the accelerated storage at 60 °C and 75% relative humidity, the moisture content of all oil powders increased during the initial period (day 0–4) and then stabilized from day 4 to day 16 (Fig. 3A). At the end of the storage period (day 16), the moisture contents of the PP, PM5, PM10, and PM15 samples increased by 2.4, 2.2, 2.0, and 2.4 times, respectively, compared with day 0. This can be attributed to the hygroscopic nature of powder materials, which readily adsorb water vapor from the surrounding environment until equilibrium moisture is reached, particularly under the storage condition of 75% relative humidity (Chaturvedi & Chakraborty, 2024). The changes in surface oil content of the powder samples are presented in Fig. 3B. The surface oil content increased continuously over the 16 days of storage. At day 16, the PP sample presented the highest surface oil content (23.59 ± 0.32 g/100 g oil), while the lowest value was observed for PM5 (21.87 ± 0.47 g/100 g oil). Throughout storage, PM5 consistently maintained the lowest surface oil content, likely due to its smoother morphology with fewer surface concavities, which effectively protected the oil core and limited oil oxidation (Fig. 1).

**Fig. 3:**
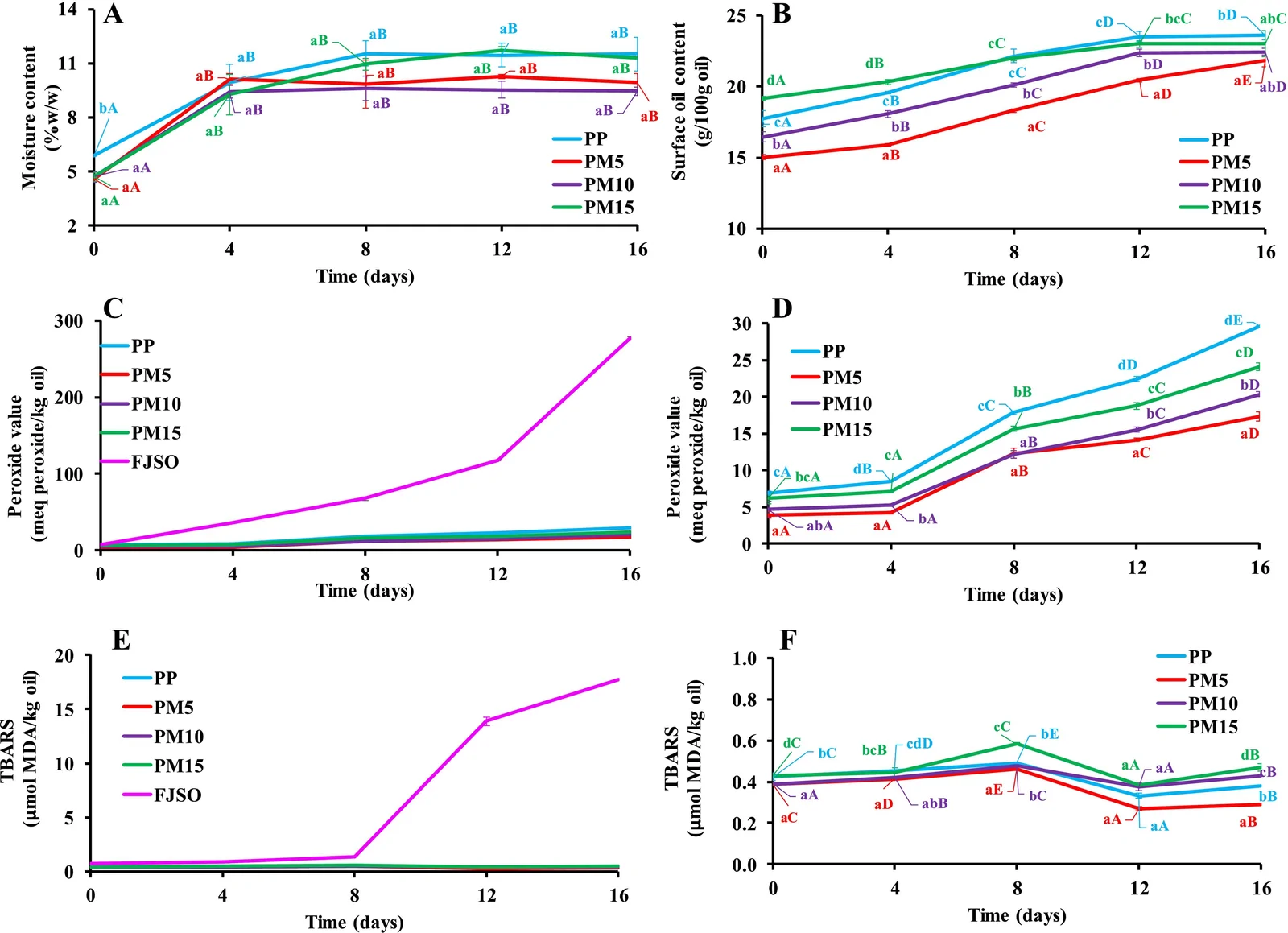
Effects of maltodextrin with varying dextrose equivalents as wall material on the moisture content (A), surface oil content (B), peroxide value (C and D), and TBARS (E and F) of *Flacourtia jangomas* seed oil encapsulation samples during accelerated storage. PP: Pea protein isolate–encapsulated *Flacourtia jangomas* seed oil; PM5: Pea protein isolate–maltodextrin (DE5) encapsulated *Flacourtia jangomas* seed oil; PM10: Pea protein isolate–maltodextrin (DE10) encapsulated *Flacourtia jangomas* seed oil; PM15: Pea protein isolate–maltodextrin (DE15) encapsulated *Flacourtia jangomas* seed oil. Abbreviations: TBARS, thiobarbituric acid reactive substances; MDA, malondialdehyde. Different lowercase letters (a-e) denote significant differences (*p* < 0.05) between the samples and uppercase letters (A-E) indicate statistically significant differences (*p* < 0.05) of samples as affected by storage days.

The peroxide value of all samples increased with storage time, reflecting the formation of hydroperoxides as primary lipid oxidation products. The non-encapsulated oil sample exhibited an extremely high peroxide value at day 16, which was approximately 8–12 times higher than that of the encapsulated samples, demonstrating the strong protective effect of encapsulation. Among the encapsulated powder samples, the PP exhibited the highest peroxide value. Xu et al. (2024) showed that mixed-wall materials often exhibit better oxidation resistance than single-wall materials, whereas microcapsules prepared from a single-wall material are less stable and exhibit lower oxidation resistance. As the DE value of maltodextrin increased, the peroxide value of the oil powder samples increased, presenting the lowest value observed in the PM5 sample, indicating that low-DE maltodextrin provided the most effective barrier against primary oil oxidation. This improved oxidative protection may be related to the longer molecular chains of low-DE maltodextrin, which can contribute to a more compact and continuous wall matrix. This interpretation is supported by the smoother and crack-free morphology of PM5 (Fig. 1) and its consistently lower surface oil content during storage (Fig. 3B). A more intact wall structure reduces the exposure of surface oil and may restrict oxygen diffusion into the encapsulating matrix, thereby limiting hydroperoxide formation.

Throughout the 16-day storage period, significantly higher TBARS values were observed in the non-encapsulated oil than in the encapsulated samples, with TBARS levels reaching approximately 37 to 60 times higher by day 16. Among the encapsulated oil powder samples, PM5 consistently exhibited the lowest TBARS values across all storage times, whereas the PP sample showed the highest values, indicating poor protection against secondary oil oxidation. As discussed by Kurek and Pratap-Singh (2020), the combination of PPI and maltodextrin plays complementary roles in forming the encapsulating matrix, thereby improving oil retention and providing greater protection against oxidation than PPI alone. The relatively higher TBARS values observed in PM15 compared with those of PM5 and PM10 suggest that maltodextrin DE also affected the protective performance of the encapsulating matrix. This may be related to differences in the molecular structure of maltodextrins with different DE values, which influence the barrier properties and consequently the oxidative stability of encapsulated oil (Zhu et al., 2022).

### 3.3 Effects of pea protein isolate and maltodextrin ratios as wall material on the *Flacourtia jangomas* seed oil encapsulation

The effects of the pea protein isolate and maltodextrin ratios on the properties of *Flacourtia jangomas* seed oil powder are shown in Table 3.

**Table 3:** Effect of pea protein isolate and maltodextrin ratio as wall material on the properties of *Flacourtia jangomas* seed oil powder.

		*Flacourtia jangomas* seed oil encapsulation samples with different ratios of pea protein isolate and maltodextrin				
		PM25	PM33	PM50	PM67	PM75
Encapsulation efficiency (%w/w)		87.8 ± 1.15ᵃ	88.11 ± 0.99ᵃ	90.82 ± 0.63ᵇ	90.65 ± 0.52ᵇ	86.4 ± 0.85ᵃ
Encapsulation yield (%w/w)		51.2 ± 2.05ᵃ	53.36 ± 1.86ᵃ	55.55 ± 1.31ᵃ	42.12 ± 1.97ᵇ	44.42 ± 1.25ᵇ
Moisture content (%w/w)		4.22 ± 0.06ᵃ	4.51 ± 0.37^a^	4.57 ± 0.32^a^	4.88 ± 0.32ᵃᵇ	4.91 ± 0.44ᵃ^b^
Mean particle size (μm)		71.0 ± 2.4^a^	85.6 ± 1.0^b^	99.1 ± 1.3^c^	104.0 ± 1.4^d^	131.0 ± 1.4^e^
Span value		2.67 ± 0.06ᵈ	2.40 ± 0.05ᶜ	2.32 ± 0.03ᶜ	2.19 ± 0.10ᵇ	1.93 ± 0.04ᵃ
Bulk density (g/cm^3^)		0.211 ± 0.003ᵃᵇ	0.233 ± 0.008ᵃᵇ	0.235 ± 0.004ᵇ	0.231 ± 0.019ᵃᵇ	0.209 ± 0.006ᵃ
Tapped density (g/cm^3^)		0.476 ± 0.000ᵃ	0.459 ± 0.059ᵃ	0.455 ± 0.001ᵃ	0.502 ± 0.035ᵃ	0.477 ± 0.000ᵃ
Carr index (%)		55.78 ± 0.47^b^	48.92 ± 3.46^a^	48.23 ± 0.61^a^	54.01 ± 0.35^b^	56.23 ± 0.91^b^
Hausner ratio		2.26 ± 0.02^b^	1.97 ± 0.13^a^	1.93 ± 0.02^a^	2.17 ± 0.02^b^	2.29 ± 0.05^b^
Color	L^⁎^	88.0 ± 0.3ᵈ	87.8 ± 0.0ᶜ	85.5 ± 0.4ᵇ	84.9 ± 0.1ᵇ	84.1 ± 0.1ᵃ
	a^⁎^	0.7 ± 0.1ᵃ	1.1 ± 0.1ᵇ	1.9 ± 0.1ᶜ	2.1 ± 0.2ᶜ	2.5 ± 0.0ᵈ
	b^⁎^	15.2 ± 0.2ᵃ	15.6 ± 0.4ᵃ	17.7 ± 0.2ᵇ	17.9 ± 0.4ᵇ	18.5 ± 0.0ᵇ

Values are means of triplicate ± standard deviation. Values followed by different letter superscripts in the same row differ significantly (*p* < 0.05). PM25; PM33; PM50; PM67; PM75: Pea protein isolate–maltodextrin encapsulated *Flacourtia jangomas* seed oil containing pea protein isolate at levels of 25%, 33%, 50%, 67%, and 75%, respectively.

The mixing ratios had a slight effect on encapsulation efficiency (*p* < 0.05). As the proportion of PPI in the carrier matrix increased from 25% to 67%, the EE rose by 3%. The highest encapsulation efficiencies were obtained at PM50 and PM67 samples, reaching 90.82% and 90.65%, respectively. However, further increases in PPI content led to a decline in EE (Table 3). At lower PPI ratios (PM25 and PM33 samples), the emulsifier concentration was insufficient to completely cover the oil droplet surface, leading to oil leakage. In contrast, a higher PPI ratio (PM75) led to the formation of brittle wall structures, increased network stiffness, making the capsules susceptible to cracking and structural shrinkage during ice crystal sublimation (Luthra et al., 2007).

The EY of FJSO did not differ significantly as the PPI content in the carrier mixture gradually increased from 25% to 50%; however, when the PPI content was further increased from 50 to 75%, the EY tended to decrease. Proteins enhance yield due to their emulsifying and film-forming properties, particularly at appropriate concentrations, where they form a protective surface layer that reduces stickiness. However, excessive protein content can reduce encapsulation yield due to the formation of aggregated and heterogeneous structures, which may increase emulsion droplet disruption and free fat release during drying (Kurtz et al., 2025). The PM50 sample showed the highest encapsulation yield (55.55%). These findings align with the trends reported by Hu et al. (2024), who utilized a pea protein-maltodextrin complex to encapsulate camellia seed oil by spray-drying.

As shown in Table 3, the moisture content of all capsule samples did not differ significantly. As the proportion of PPI in the carrier mixture rose from 25% to 75%, the average particle size of the oil powder increased by 1.8-fold. Increasing the PPI ratio increased the emulsion viscosity, thereby leading to larger emulsion droplets and freeze-dried powder particles, with additional particle enlargement due to protein agglomeration (Olsmats & Rennie, 2024). The span index decreased as the PPI ratio in the wall material increased. As protein concentration increases, dispersed oil droplets are more effectively encapsulated, thereby reducing interfacial tension and inhibiting coalescence during emulsification (Le Flem et al., 2026).

Increasing the proportion of PPI in the wall material did not significantly affect the bulk density or tapped density of the oil powder samples (Table 3). Notably, CI and HR showed a consistent trend, decreasing to a minimum at intermediate pea protein levels (33–50%) and increasing thereafter, suggesting that moderate protein content was more favorable for powder flowability than either lower or higher protein concentrations. Powders with high protein content are particularly susceptible to interparticle interactions, leading to increased cohesive forces in the bulk powder and reduced flowability (Hazlett et al., 2021) Samples containing higher levels of PPI exhibited slightly lower L* values, which may be attributed to the characteristic dark yellow color of PPI, leading to a reduction in powder brightness. Consistent with this observation, the a* and b* values increased slightly at high PPI levels, indicating enhanced redness and yellowness.

Scanning electron microscopy (SEM) images of FJSO powder samples with different ratios of PPI and maltodextrin as wall material are presented in Fig. 4. Freeze-drying produced microcapsules with irregular, porous, and flaky morphologies with a mean particle size from 71.0 ± 2.4 to 131.0 ± 1.4 μm when the ratio of PPI increased from 25 to 75% (Table 3). Furthermore, the morphological characteristics of the microcapsules were crucially affected by the mixing ratio of PPI and maltodextrin. As the protein concentration in the carrier matrix increased, particles with rougher and more angular surfaces were formed (PM67 and PM75). Additionally, a higher protein content tended to promote aggregation of microcapsules into clusters rather than remaining discrete, due to electrostatic interactions and hydrogen bonding between protein molecules (Gharsallaoui et al., 2012). According to Vélez-Erazo et al. (2021), excessively high protein ratios can reduce membrane formation because increased protein-protein interactions promote aggregation and rigid structure formation, cause morphological instability, and decrease the effectiveness of core protection in oil microcapsule systems.

**Fig. 4:**
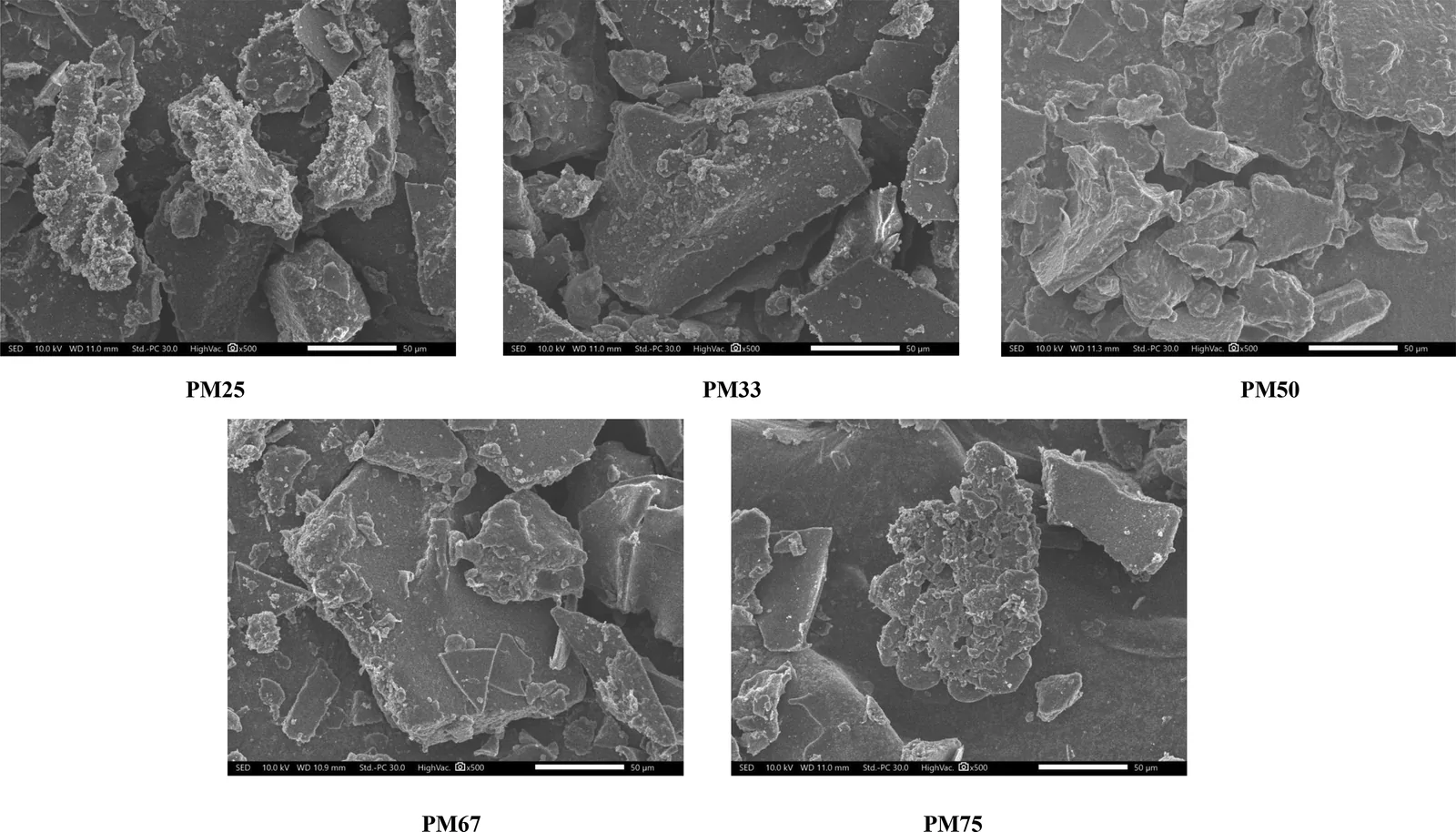
Scanning electron microscopy (SEM) of *Flacourtia jangomas* seed oil encapsulation samples with different ratios of pea protein isolate and maltodextrin as wall material. PM25; PM33; PM50; PM67; PM75: Pea protein isolate–maltodextrin encapsulated *Flacourtia jangomas* seed oil containing pea protein isolate at levels of 25%, 33%, 50%, 67%, and 75%, respectively.

Please insert Fig. 5 here.

**Fig. 5:**
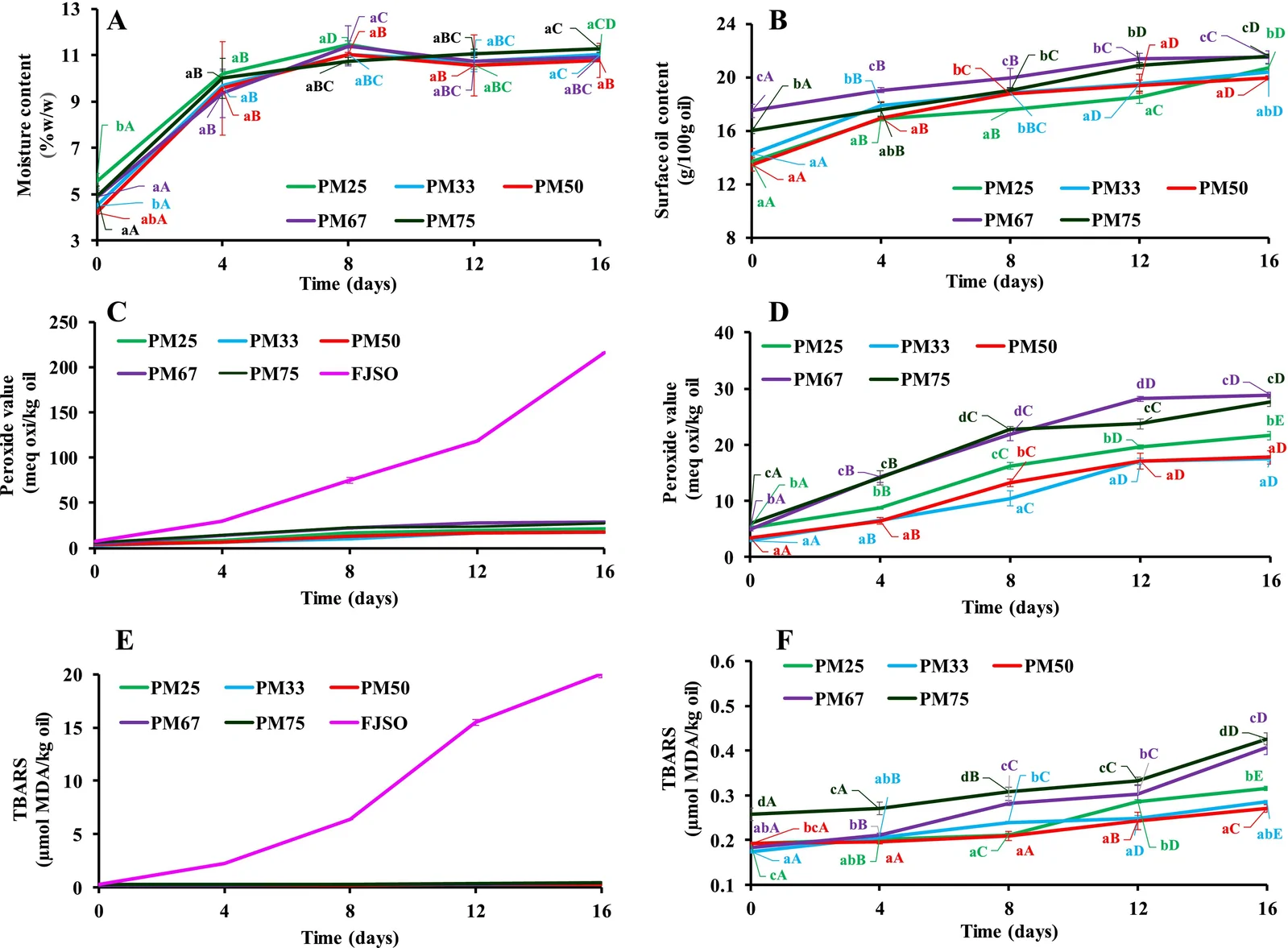
Effect of pea protein isolate and maltodextrin ratio as wall material on the moisture content (A), surface oil content (B), peroxide value (C, D), and TBARS (E, F) of *Flacourtia jangomas* seed oil encapsulation samples during accelerated storage. PM25; PM33; PM50; PM67; PM75: Pea protein isolate-maltodextrin encapsulated *Flacourtia jangomas* seed oil containing pea protein isolate at levels of 25%, 33%, 50%, 67%, and 75%, respectively. Abbreviations:TBARS: thiobarbituric acid reactive substances; MDA: malondialdehyde. Different lowercase letters (a-e) denote significant differences (*p* < 0.05) between the samples and uppercase letters (A-E) indicate statistically significant differences (*p* < 0.05) of samples as affected by storage days.

Under accelerated storage conditions, the moisture content of the encapsulated oil powder increased rapidly within the first 8 days. However, no statistically significant difference was observed between day 12 and day 16 (Fig. 5A). The surface oil content of the oil powder samples gradually increased throughout the storage period. The PM50 sample exhibited the lowest surface oil content, which increased 1.5-fold after 16 days of accelerated storage (Fig. 5B). In contrast, samples with higher PPI levels (67% and 75%) showed significantly higher surface oil contents, exceeding 21 g/100 g oil at the end of storage. This suggests that a balanced ratio of PP and maltodextrin could form a compact and effective encapsulating matrix that limits oil migration to the particle surface, whereas protein-rich formulations (67% and 75%) tend to form rigid films prone to shrinkage and cracking during freeze-drying, thereby promoting oil leakage.

Fig. 5C illustrates the variation in peroxide value of FJSO during accelerated storage. For all oil powder samples, the peroxide value gradually increased during storage due to lipid oxidation. Notably, the unencapsulated oil exhibited a markedly higher rate of increase in peroxide value. By day 16, the peroxide value of the unencapsulated oil reached 216.30 ± 0.18 meq of peroxide/kg oil, approximately 12 times that of PM50, clearly demonstrating the protective effect of the encapsulating matrix. On the 16th day of storage, samples containing 33% (PM33) and 50% (PM50) PPI exhibited the lowest peroxide values, at 17.81 ± 1.21 and 17.50 ± 0.48 meq peroxide/kg oil, respectively. In contrast, protein-rich formulations (PM67 and PM75) showed significantly higher peroxide values, reaching 28.87 ± 0.50 and 27.65 ± 0.78 meq peroxide/kg oil, respectively. The higher peroxide values of the protein-rich formulations may be associated with their less uniform and more aggregated structures (Fig. 4) and higher surface oil contents (Fig. 5B), which provide a less effective physical barrier and result in higher surface oil exposure, thereby increasing the susceptibility of the oil to primary oxidation.

The secondary oxidation index (TBARS) of the non-encapsulated oil increased by 71 times over 16 days of storage, whereas that of the oil powder samples increased by only 1.4 to 2.2 times. Samples with higher PPI ratios of 67% and 75% exhibited higher TBARS values, reaching 0.407 ± 0.016 and 0.426 ± 0.013 μmol MDA/kg oil, respectively. This may be attributed to the limited emulsifying capacity at excessive protein concentrations. The appropriate PPI ratios improve emulsion stability (Premi & Sharma, 2017) and enhance oxidative stability. The superior performance of the 50% ratio can be attributed to the balanced contribution of PPI and maltodextrin, which together form a dense, stable coating with improved oxygen-barrier properties.

## 4 Conclusion

*Flacourtia jangomas* seed oil demonstrated considerable potential as a functional ingredient due to its high content of unsaturated fatty acids and strong antioxidant activity. This study showed that both the DE of maltodextrin and the PPI proportion influenced the EE, EY, physicochemical properties, and oxidative stability of freeze-dried FJSO capsules. Among the formulations investigated, maltodextrin with DE 5 and a PPI of 50% in the carrier matrix was the most suitable, as evidenced by its favorable encapsulation performance and effective protection against lipid oxidation under accelerated storage conditions. These findings provide insight into the complementary functions of protein and carbohydrate wall materials in controlling the physicochemical properties and protective performance of encapsulated oils. The conversion of *Flacourtia jangomas* seed oil into a stable powder may also facilitate its handling, storage, and incorporation into functional food formulations. However, oxidative stability was evaluated only under accelerated storage conditions, and the performance of the developed powder in actual food systems remains unknown. Further studies should therefore evaluate its long-term stability under conventional storage conditions, assess its behavior in different food matrices, and investigate process scale-up to establish its practical applicability.

## CRediT authorship contribution statement

**Thi Thuy Le:** Writing – original draft, Methodology, Investigation, Formal analysis, Data curation. **Phuong Vy Tra:** Investigation, Formal analysis, Data curation. **Ngoc Nhu Huynh Nguyen:** Investigation, Formal analysis, Data curation. **Hoang Nhi Tran:** Investigation, Formal analysis, Data curation. **Thi Thu Tra Tran:** Visualization, Validation, Supervision, Resources. **Van Viet Man Le:** Writing – review & editing, Supervision, Project administration, Conceptualization.

## Declaration of competing interest

The authors declare that they have no known competing financial interests or personal relationships that could have appeared to influence the work reported in this paper.

## Data Availability

Data will be made available on request.
